# Intravascular lymphoma presenting as a specific pulmonary embolism and acute respiratory failure: a case report

**DOI:** 10.1186/1752-1947-3-7253

**Published:** 2009-05-12

**Authors:** Sophie Georgin-Lavialle, Michael Darmon, Lionel Galicier, Marinos Fysekidis, Elie Azoulay

**Affiliations:** 1 AP-HP, Hôpital Saint-Louis, Medical ICU, Department of Immunology and Department of Nuclear Médicine, 75010 Paris, France; 2 Medical Intensive Care Unit, Saint-Louis University Hospital, 1 Avenue Claude Vellefaux, 75010 Paris, France

## Abstract

**Introduction:**

The occurrence of an intravascular lymphoma with severe pulmonary involvement mimicking pulmonary embolism is described.

**Case presentation:**

A 38-year-old man was referred to our intensive care unit with acute respiratory failure and long lasting fever. Appropriate investigations failed to demonstrate any bacterial, viral, parasitic or mycobacterial infection. A chest computed tomography scan ruled out any proximal or sub-segmental pulmonary embolism but the ventilation/perfusion lung scan concluded that there was a high probability of pulmonary embolism. The cutaneous biopsy pathology diagnosed intravascular lymphoma.

**Conclusion:**

Intravascular lymphoma is a rare disease characterized by exclusive or predominant growth of neoplastic cells within the lumina of small blood vessels. Lung involvement seems to be common, but predominant lung presentation of this disease is rare. In our patient, urgent chemotherapy, along with adequate supportive care allowed complete recovery.

## Introduction

Intravascular lymphoma (IVL) is an intravascular proliferation of clonal lymphocytes with little to no parenchymal involvement. The clinical presentation is highly variable, ranging from no or limited organ involvement to multiple organ failure. Therefore, the diagnosis is often difficult. Proliferation of lymphoma cells in blood vessels of parenchymal organs results in vessel obliteration and ischemia. We report a patient with an intravascular lymphoma with predominant pulmonary involvement, presenting as acute respiratory failure and a specific pulmonary embolism.

## Case presentation

In May 2005, a 38-year-old man was referred to our intensive care unit with acute respiratory failure and long lasting fever. He had an unremarkable medical history until 5 months ago when he was referred for a 15 kg weight loss and fever. Clinical examination was normal at that time. Appropriate investigations failed to demonstrate any bacterial, viral, parasitic or mycobacterial infection, including intracellular bacteria, HIV, hepatitis B virus, hepatitis C virus, tuberculosis, typhoid, syphilis, or brucellosis. Antinuclear antibodies, rheumatoid factors, and anti-neutrophil cytoplasmic antibodies were also negative. No monoclonal immunoglobulin was detected in serum or urine. Thoracic and abdominal computed tomography (CT) scan revealed no abnormality except a homogeneous splenomegaly. In April 2005, bone marrow aspiration demonstrated evidence of hemophagocytosis without any other abnormality and a lymphoproliferation was therefore suspected. The bone marrow biopsy was normal and diagnostic splenectomy was performed, but the pathologic examination of the spleen was inconclusive. Since there was a high suspicion of histiocytic lymphohistiocytosis and of lymphoproliferation, steroids were started leading to transient improvement in the patient's clinical condition.

In May 2005, the patient developed acute respiratory failure, with profound hypoxemia of PaO_2_ 35 mmHg while breathing room air, and a shunt effect only partly corrected by oxygen PaO_2_ 51 mmHg and PaCO_2_ 25 mmHg while under 5 L/minute O_2_. He was referred to the ICU with a clinical diagnosis of highly probable pulmonary embolism with acute right heart insufficiency.

In addition to persistent fever (39.3°C), clinical examination revealed an exanthema, as well as jugular venous distension, without any other signs of cardiac failure. The clinical examination was otherwise unremarkable. Clinical and radiological examination showed no evidence of pneumonia or bronchiolitis. Ultrasonography of the deep leg veins was negative. His hemoglobin level was 10.6 g/dL mean corpuscular volume, 94 fL; mean corpuscular hemoglobin 31.2 pg, and platelet count was 117 g/L. The peripheral blood picture was unremarkable and the serum lactate dehydrogenase level was elevated at 1164 U/L.

**Figure 1:**
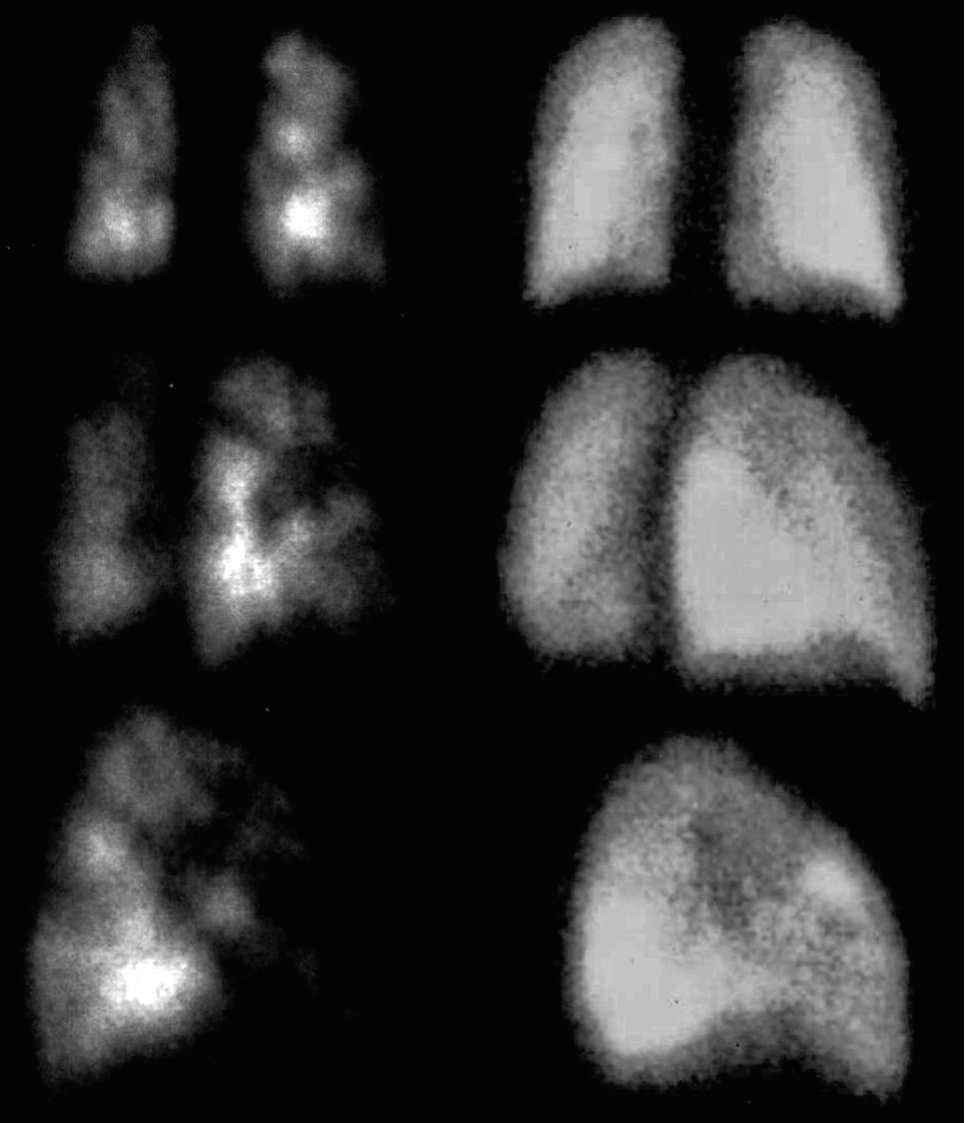
**Perfusion lung scan before (left panel) and after (right panel) cancer chemotherapy**.

**Figure 2:**
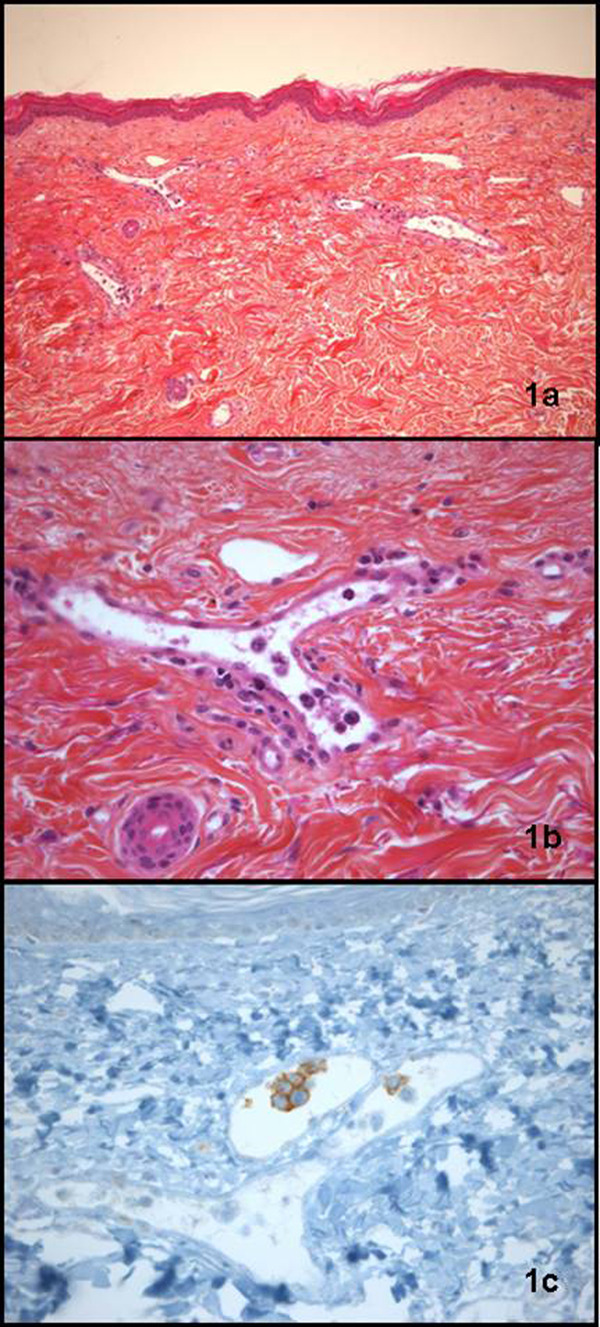
**Cutaneous punch biopsy showing an intravascular lymphocytic infiltrate **(a)** composed of large atypical cells **(b)** that were CD20 positive on an immunohistochemical stain **(c)****.

A chest CT-scan ruled out any proximal or sub-segmental pulmonary embolism but the ventilation/perfusion lung scan indicated a high probability of pulmonary embolism with multiple bilateral and distal perfusion defects without evidence of a right-left shunt (Figure 1, left panel). A cutaneous biopsy was performed, showing several abnormal lymphocytes on cytological examination of biopsy smears and suggesting lymphoma. The cutaneous biopsy pathology revealed large pleomorphic cells expressing CD20 in blood vessels and was diagnosed as intravascular lymphoma (Figure 2).

Despite the acute respiratory failure and the profound hypoxemia, non-invasive mechanical ventilation was not performed mainly because of the acute right insufficiency. The patient required no organ support other than oxygen therapy.

Urgent cancer chemotherapy of cyclophosphamide, adriamycin, VP16 and steroids (CHOP) was started in order to treat the malignant pulmonary vascular involvement. This treatment led to a dramatic improvement in the patient's clinical condition. Five days after admission, he was free of oxygen therapy and the perfusion lung scan was normal (Figure 1, right panel). After six cycles of chemotherapy with CHOP-Methotrexate associated with Rituximab, the patient was considered to be in complete remission (CR). High-dose chemotherapy with autologous stem cell transplantation was carried out. The patient was alive in CR at 1 June 2008.

## Discussion

Intravascular large B-cell lymphoma is a rare disease characterized by exclusive or predominant growth of neoplastic cells within the lumina of small blood vessels [1]. Proliferation of lymphoma cells in blood vessels of parenchymal organs results in vessel obliteration and ischemia. Less than half of patients have antemortem diagnosis. Lung involvement seems to be common, but predominant lung presentation of this disease is rare [2,3]. To the best of our knowledge, only four cases have been reported with pulmonary hypertension or suspicion of pulmonary embolism [2,4,5]. Anthracycline-based chemotherapy has been shown to be associated with a nearly 60% response rate and a 3-year overall survival rate higher than 30%. Therefore CHOP and CHOP-like regimens are considered to be efficient [6]. Fever of unknown origin is the presenting feature in 60% of intravascular lymphoma and is frequently associated with a hemophagocytic lymphohistiocytosis [6]. Although our patient did not meet all the usual criteria for this syndrome, the clinical presentation of bicytopenia, long lasting fever, and splenomegaly, along with the typical image of hemophagocytosis at bone marrow aspiration in this context allowed us to suspect hemophagocytic lymphohistiocytosis and was therefore highly suggestive of lymphoma [6,7]. Pulmonary involvement of hemophagocytic lymphohistiocytosis may occur in up to 30% of patients but is usually associated with pulmonary infiltrates [8]. The clinical course of the disease, negativity of the extensive search for acute and chronic pulmonary infection and the absence of pneumonia or bronchiolitis allowed us to rule out an infectious disease. Lastly, the high probability ventilation/perfusion lung scan with a normal CT-scan, the absence of deep vein thrombosis along with the presence of abnormal lymphocytes led to the diagnosis of non-thrombotic pulmonary embolism and to initiate urgent cancer chemotherapy for malignant vascular pulmonary involvement.

In patients with specific organ failure and a high suspicion of active malignancy, urgent chemotherapy, along with adequate supportive care is the only means of ensuring recovery. In a recent series of selected patients, we report about 50% survival in cancer patients with specific organ failure and the need of immediate cancer chemotherapy initiation, along with mechanical ventilation, vasopressors and dialysis [9].

## Conclusion

This case confirms that, although rare, intravascular lymphoma may be responsible for precapillary pulmonary arterial hypertension and may cause life-threatening manifestations requiring urgent chemotherapy. Cutaneous biopsy may provide a diagnosis and should be performed on an emergency basis each time a diagnosis of intravascular lymphoma is suspected. In the sickest patients with life-threatening complications, the high suspicion of malignancy along with the presence of abnormal cells should prompt the clinician to initiate appropriate cancer therapy before definite diagnosis in order to restore organ function and increase the chances of survival.

## List of abbreviations

CR: complete remission; CT: computed tomography; IVL: intravascular lymphomas.

## Consent

Written informed consent was obtained from the patient for publication of this case report and any accompanying images. A copy of the written consent is available for review by the Editor-in-Chief of this journal.

## Competing interests

The authors declare that they have no competing interests.

## Authors' contributions

MD and SGL acquired the data and drafted the manuscript. EA critically revised the manuscript and supervised the work. LG was the attending hematologist. The perfusion lung-scan was performed by MF.
